# 1-Chloro-1-[(*Z*)-2-phenyl­hydrazin-1-yl­idene]propan-2-one

**DOI:** 10.1107/S1600536812028759

**Published:** 2012-06-30

**Authors:** Hatem A. Abdel-Aziz, Tze Shyang Chia, Hoong-Kun Fun

**Affiliations:** a Department of Pharmaceutical Chemistry, College of Pharmacy, King Saud University, PO Box 2457, Riyadh 11451, Saudi Arabia; b X-ray Crystallography Unit, School of Physics, Universiti Sains Malaysia, 11800 USM, Penang, Malaysia

## Abstract

The title compound, C_9_H_9_ClN_2_O, is close to planar (r.m.s. deviation for the non-H atoms = 0.0446 Å); it exists in a *cis* conformation with respect to the C=N double bond. In the crystal, the ketone O atom accepts both N—H⋯O and C—H⋯O hydrogen bonds, which leads to [010] infinite chains incorporating *R*_2_^1^(6) loops. The crystal structure also features a C—H⋯π inter­action.

## Related literature

For synthetic applications of hydrazonoyl chlorides, see: Abdel-Aziz & Mekawey (2009 ▶). For graph-set descriptors of hydrogen-bond motifs, see: Bernstein *et al.* (1995 ▶). For related structures. see: Asiri *et al.* (2011*a* ▶,*b* ▶). For a historical perspective on the synthesis, see: Dieckmann & Platz (1905 ▶). For the stability of the temperature controller used in the data collection, see: Cosier & Glazer (1986 ▶).
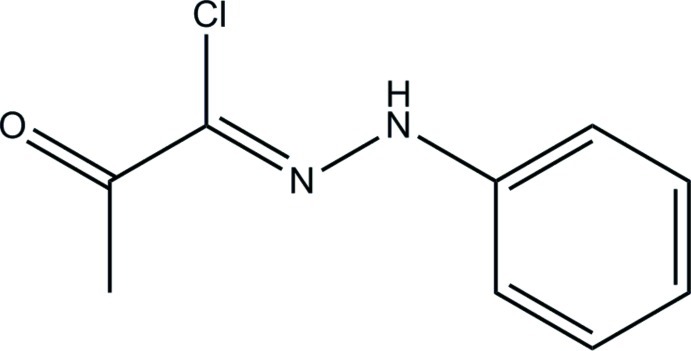


## Experimental

### 

#### Crystal data

C_9_H_9_ClN_2_O
*M* *_r_* = 196.63
Monoclinic, 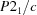

*a* = 7.2681 (14) Å
*b* = 12.361 (2) Å
*c* = 10.704 (2) Å
β = 101.158 (3)°
*V* = 943.5 (3) Å^3^
*Z* = 4
Mo *K*α radiation
μ = 0.36 mm^−1^
*T* = 100 K
0.37 × 0.21 × 0.10 mm

#### Data collection

Bruker APEX DUO CCD diffractometer
Absorption correction: multi-scan (*SADABS*; Bruker, 2009 ▶) *T*_min_ = 0.877, *T*_max_ = 0.963
8906 measured reflections
2722 independent reflections
2225 reflections with *I* > 2σ(*I*)
*R*_int_ = 0.030

#### Refinement

*R*[*F* ^2^ > 2σ(*F* ^2^)] = 0.049
*wR*(*F* ^2^) = 0.148
*S* = 1.06
2722 reflections
124 parameters
H atoms treated by a mixture of independent and constrained refinement
Δρ_max_ = 0.86 e Å^−3^
Δρ_min_ = −0.37 e Å^−3^

### 

Data collection: *APEX2* (Bruker, 2009 ▶); cell refinement: *SAINT* (Bruker, 2009 ▶); data reduction: *SAINT*; program(s) used to solve structure: *SHELXTL* (Sheldrick, 2008 ▶); program(s) used to refine structure: *SHELXTL*; molecular graphics: *SHELXTL*; software used to prepare material for publication: *SHELXTL* and *PLATON* (Spek, 2009 ▶).

## Supplementary Material

Crystal structure: contains datablock(s) global, I. DOI: 10.1107/S1600536812028759/hb6870sup1.cif

Structure factors: contains datablock(s) I. DOI: 10.1107/S1600536812028759/hb6870Isup2.hkl

Supplementary material file. DOI: 10.1107/S1600536812028759/hb6870Isup3.cml

Additional supplementary materials:  crystallographic information; 3D view; checkCIF report

## Figures and Tables

**Table 1:** Hydrogen-bond geometry (Å, °) *Cg*1 is the centroid of the C1–C6 ring.

*D*—H⋯*A*	*D*—H	H⋯*A*	*D*⋯*A*	*D*—H⋯*A*
N1—H1*N*1⋯O1^i^	0.99 (3)	2.01 (3)	2.948 (2)	157 (2)
C1—H1*A*⋯O1^i^	0.95	2.45	3.237 (3)	140
C9—H9*B*⋯*Cg*1^ii^	0.98	2.68	3.560 (2)	149

Symmetry codes: (i) 
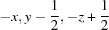
; (ii) 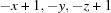.

**Fig. 1.**
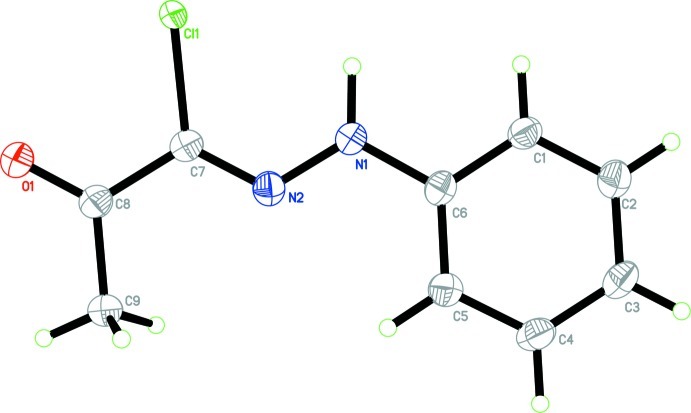
The molecular structure of the title compound with 50% probability displacement ellipsoids.

**Fig. 2.**
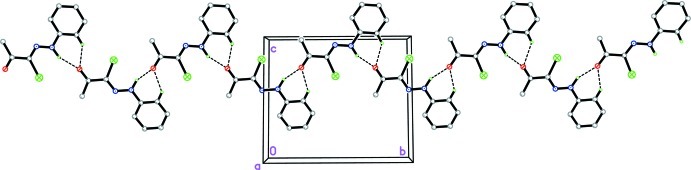
The crystal packing of the title compound. The dashed lines represent the hydrogen bonds. For clarity sake, hydrogen atoms not involved in hydrogen bonding have been omitted.

## supplementary crystallographic information

### Comment

As part of our ongoing studies of the synthetic chemistry of hydrazonoyl chlorides (Abdel-Aziz & Mekawey, 2009), the title compound was prepared and its crystal structure is now reported.

The asymmetric unit of the title compound is shown in Fig. 1. All of the non-H atoms lie nearly on a plane with r.m.s. deviation of 0.0446 Å. The molecule exists in *cis* configuration with respect to the C7═N2 double bond. Bond lengths and angles are comparable to those in related structures (Asiri *et al.*, 2011*a*,*b*).

In the crystal (Fig. 2), molecules are linked by N1—H1N1···O1 and C1—H1A···O1 hydrogen bonds (Table 1), generating *R*_2_^1^(6) loops (Bernstein *et al.*, 1995) and forming infinite wave-like chains along [010]. The packing also features a C—H···π interaction (Table 1), involving *Cg*1, which is the centroid of C1–C6 ring.

### Experimental

The title compound was prepared by the coupling reaction of 3-chloro-2,4-pentanedione and the diazonium salt of aniline at 0–5 °C (Dieckmann & Platz, 1905). Yellow blocks were recrystallised from ethanol solution.

### Refinement

The atom H1N1 was located in a difference fourier map and refined freely [N1—H1N1 = 1.00 (3) Å]. The remaining H atoms were positioned geometrically [C—H = 0.95 and 0.98 Å] and refined using a riding model with *U*_iso_(H) = 1.2 or 1.5*U*_eq_(C). A rotating group model was applied to the methyl group. Five outliers, (102), (213), (113), (315) and (011) were omitted in the final refinement.

### Crystal data

**Table:**

C_9_H_9_ClN_2_O	*F*(000) = 408
*M**_r_* = 196.63	*D*_x_ = 1.384 Mg m^−^^3^
Monoclinic, *P*2_1_/*c*	Mo *K*α radiation, λ = 0.71073 Å
Hall symbol: -P 2ybc	Cell parameters from 3613 reflections
*a* = 7.2681 (14) Å	θ = 2.5–30.0°
*b* = 12.361 (2) Å	µ = 0.36 mm^−^^1^
*c* = 10.704 (2) Å	*T* = 100 K
β = 101.158 (3)°	Block, yellow
*V* = 943.5 (3) Å^3^	0.37 × 0.21 × 0.10 mm
*Z* = 4	

### Data collection

**Table:**

Bruker APEX DUO CCD diffractometer	2722 independent reflections
Radiation source: fine-focus sealed tube	2225 reflections with *I* > 2σ(*I*)
Graphite monochromator	*R*_int_ = 0.030
φ and ω scans	θ_max_ = 30.0°, θ_min_ = 2.9°
Absorption correction: multi-scan (*SADABS*; Bruker, 2009)	*h* = −10→10
*T*_min_ = 0.877, *T*_max_ = 0.963	*k* = −17→14
8906 measured reflections	*l* = −15→13

### Refinement

**Table:**

Refinement on *F*^2^	Secondary atom site location: difference Fourier map
Least-squares matrix: full	Hydrogen site location: inferred from neighbouring sites
*R*[*F*^2^ > 2σ(*F*^2^)] = 0.049	H atoms treated by a mixture of independent and constrained refinement
*wR*(*F*^2^) = 0.148	*w* = 1/[σ^2^(*F*_o_^2^) + (0.0802*P*)^2^ + 0.7657*P*] where *P* = (*F*_o_^2^ + 2*F*_c_^2^)/3
*S* = 1.06	(Δ/σ)_max_ < 0.001
2722 reflections	Δρ_max_ = 0.86 e Å^−^^3^
124 parameters	Δρ_min_ = −0.37 e Å^−^^3^
0 restraints	Extinction correction: *SHELXTL* (Sheldrick, 2008), Fc^*^=kFc[1+0.001xFc^2^λ^3^/sin(2θ)]^-1/4^
Primary atom site location: structure-invariant direct methods	Extinction coefficient: 0.008 (3)

### Special details

**Table:**

Experimental. The crystal was placed in the cold stream of an Oxford Cryosystems Cobra open-flow nitrogen cryostat (Cosier & Glazer, 1986) operating at 100.0 (1) K.
Geometry. All e.s.d.'s (except the e.s.d. in the dihedral angle between two l.s. planes) are estimated using the full covariance matrix. The cell e.s.d.'s are taken into account individually in the estimation of e.s.d.'s in distances, angles and torsion angles; correlations between e.s.d.'s in cell parameters are only used when they are defined by crystal symmetry. An approximate (isotropic) treatment of cell e.s.d.'s is used for estimating e.s.d.'s involving l.s. planes.
Refinement. Refinement of *F*^2^ against ALL reflections. The weighted *R*-factor *wR* and goodness of fit *S* are based on *F*^2^, conventional *R*-factors *R* are based on *F*, with *F* set to zero for negative *F*^2^. The threshold expression of *F*^2^ > σ(*F*^2^) is used only for calculating *R*-factors(gt) *etc*. and is not relevant to the choice of reflections for refinement. *R*-factors based on *F*^2^ are statistically about twice as large as those based on *F*, and *R*- factors based on ALL data will be even larger.

### Fractional atomic coordinates and isotropic or equivalent isotropic displacement parameters (Å^2^)

**Table:**

	*x*	*y*	*z*	*U*_iso_*/*U*_eq_	
Cl1	−0.00357 (7)	0.01567 (4)	0.18248 (4)	0.02310 (16)	
O1	0.0096 (2)	0.24567 (12)	0.25220 (13)	0.0275 (3)	
N1	0.1947 (2)	−0.08946 (14)	0.41978 (16)	0.0235 (4)	
N2	0.1820 (2)	0.01726 (13)	0.42826 (16)	0.0220 (3)	
C1	0.2872 (3)	−0.26204 (17)	0.51547 (19)	0.0230 (4)	
H1A	0.2243	−0.2962	0.4397	0.028*	
C2	0.3780 (3)	−0.32397 (18)	0.6175 (2)	0.0265 (4)	
H2A	0.3769	−0.4006	0.6112	0.032*	
C3	0.4705 (3)	−0.27448 (19)	0.72882 (19)	0.0278 (4)	
H3A	0.5321	−0.3169	0.7985	0.033*	
C4	0.4715 (3)	−0.16205 (19)	0.7370 (2)	0.0277 (4)	
H4A	0.5349	−0.1281	0.8128	0.033*	
C5	0.3816 (3)	−0.09862 (18)	0.63614 (19)	0.0250 (4)	
H5A	0.3831	−0.0220	0.6428	0.030*	
C6	0.2890 (3)	−0.14930 (17)	0.52499 (18)	0.0216 (4)	
C7	0.0985 (3)	0.07370 (17)	0.33405 (18)	0.0225 (4)	
C8	0.0876 (3)	0.19245 (16)	0.34351 (18)	0.0221 (4)	
C9	0.1800 (3)	0.24284 (17)	0.46791 (19)	0.0256 (4)	
H9A	0.1471	0.3197	0.4678	0.038*	
H9B	0.3164	0.2353	0.4787	0.038*	
H9C	0.1367	0.2063	0.5382	0.038*	
H1N1	0.129 (4)	−0.130 (2)	0.344 (3)	0.032 (7)*	

### Atomic displacement parameters (Å^2^)

**Table:**

	*U*^11^	*U*^22^	*U*^33^	*U*^12^	*U*^13^	*U*^23^
Cl1	0.0330 (3)	0.0140 (2)	0.0192 (2)	0.00383 (16)	−0.00273 (17)	−0.00116 (15)
O1	0.0323 (8)	0.0238 (7)	0.0241 (7)	0.0020 (6)	−0.0001 (6)	0.0020 (6)
N1	0.0275 (8)	0.0211 (8)	0.0202 (8)	0.0017 (6)	0.0006 (6)	0.0010 (6)
N2	0.0214 (7)	0.0211 (8)	0.0235 (8)	0.0000 (6)	0.0040 (6)	0.0013 (6)
C1	0.0220 (9)	0.0246 (10)	0.0210 (8)	−0.0009 (7)	0.0010 (7)	0.0004 (7)
C2	0.0271 (9)	0.0252 (10)	0.0268 (10)	0.0011 (8)	0.0041 (8)	0.0040 (8)
C3	0.0270 (10)	0.0324 (11)	0.0222 (9)	0.0008 (8)	0.0000 (7)	0.0064 (8)
C4	0.0280 (10)	0.0326 (11)	0.0206 (9)	−0.0028 (8)	−0.0005 (7)	0.0000 (8)
C5	0.0274 (9)	0.0234 (10)	0.0234 (9)	−0.0017 (7)	0.0027 (7)	0.0002 (7)
C6	0.0207 (8)	0.0234 (9)	0.0208 (9)	0.0004 (7)	0.0040 (7)	0.0035 (7)
C7	0.0237 (9)	0.0233 (10)	0.0196 (8)	0.0003 (7)	0.0019 (7)	−0.0002 (7)
C8	0.0211 (8)	0.0240 (10)	0.0212 (9)	0.0000 (7)	0.0039 (7)	0.0005 (7)
C9	0.0283 (10)	0.0240 (10)	0.0227 (9)	−0.0004 (7)	0.0002 (7)	−0.0018 (7)

### Geometric parameters (Å, º)

**Table:**

Cl1—C7	1.798 (2)	C3—C4	1.392 (3)
O1—C8	1.223 (2)	C3—H3A	0.9500
N1—N2	1.327 (2)	C4—C5	1.391 (3)
N1—C6	1.409 (2)	C4—H4A	0.9500
N1—H1N1	1.00 (3)	C5—C6	1.397 (3)
N2—C7	1.279 (3)	C5—H5A	0.9500
C1—C2	1.392 (3)	C7—C8	1.475 (3)
C1—C6	1.397 (3)	C8—C9	1.505 (3)
C1—H1A	0.9500	C9—H9A	0.9800
C2—C3	1.391 (3)	C9—H9B	0.9800
C2—H2A	0.9500	C9—H9C	0.9800
			
N2—N1—C6	119.79 (17)	C4—C5—H5A	120.5
N2—N1—H1N1	121.9 (15)	C6—C5—H5A	120.5
C6—N1—H1N1	118.0 (15)	C5—C6—C1	120.37 (18)
C7—N2—N1	121.15 (18)	C5—C6—N1	121.67 (18)
C2—C1—C6	119.68 (19)	C1—C6—N1	117.96 (18)
C2—C1—H1A	120.2	N2—C7—C8	120.84 (18)
C6—C1—H1A	120.2	N2—C7—Cl1	122.96 (16)
C3—C2—C1	120.5 (2)	C8—C7—Cl1	116.16 (14)
C3—C2—H2A	119.8	O1—C8—C7	120.24 (18)
C1—C2—H2A	119.8	O1—C8—C9	122.88 (19)
C2—C3—C4	119.25 (19)	C7—C8—C9	116.87 (17)
C2—C3—H3A	120.4	C8—C9—H9A	109.5
C4—C3—H3A	120.4	C8—C9—H9B	109.5
C5—C4—C3	121.2 (2)	H9A—C9—H9B	109.5
C5—C4—H4A	119.4	C8—C9—H9C	109.5
C3—C4—H4A	119.4	H9A—C9—H9C	109.5
C4—C5—C6	119.0 (2)	H9B—C9—H9C	109.5
			
C6—N1—N2—C7	179.46 (17)	N2—N1—C6—C5	−4.6 (3)
C6—C1—C2—C3	0.0 (3)	N2—N1—C6—C1	175.28 (17)
C1—C2—C3—C4	−0.1 (3)	N1—N2—C7—C8	−178.98 (17)
C2—C3—C4—C5	0.2 (3)	N1—N2—C7—Cl1	−1.3 (3)
C3—C4—C5—C6	−0.1 (3)	N2—C7—C8—O1	179.02 (17)
C4—C5—C6—C1	−0.1 (3)	Cl1—C7—C8—O1	1.2 (2)
C4—C5—C6—N1	179.75 (18)	N2—C7—C8—C9	0.5 (3)
C2—C1—C6—C5	0.1 (3)	Cl1—C7—C8—C9	−177.33 (13)
C2—C1—C6—N1	−179.73 (17)		

### Hydrogen-bond geometry (Å, º)

Cg1 is the centroid of the C1–C6 ring.

**Table:**

*D*—H···*A*	*D*—H	H···*A*	*D*···*A*	*D*—H···*A*
N1—H1*N*1···O1^i^	0.99 (3)	2.01 (3)	2.948 (2)	157 (2)
C1—H1*A*···O1^i^	0.95	2.45	3.237 (3)	140
C9—H9*B*···*Cg*1^ii^	0.98	2.68	3.560 (2)	149

Symmetry codes: (i) −*x*, *y*−1/2, −*z*+1/2; (ii) −*x*+1, −*y*, −*z*+1.
